# When it could have been prevented: A maternal death from postpartum hemorrhage due to genital tract lesions complicated by urinary fistula in an Ebola treatment unit in DR Congo

**DOI:** 10.1186/s12884-026-09271-2

**Published:** 2026-06-23

**Authors:** Prince Imani-Musimwa, Emilie Grant, Daniel Mukadi-Bamuleka, Zacharie Tsongo-Kibendelwa, Rigo Fraterne-Muhayangabo, Placide Mbala-Kingebeni, Richard Kitenge-Omasumbu, Théophile Barhwamire-Kabesha, Olivier Nyakio-Ngeleza, Juakali Sihali-Kyolov, Micheline Feza-Malira, Baudouin Manwa-Budwaga, Desiré Alumeti-Munyali, Richard Bitwe-Mihanda, Dieudonné Sengey-Mushengezi-Amani, Mija Ververs

**Affiliations:** 1 Department of Obstetrics and Gynecology, School of Medicine and Public Health, University of Goma, Goma, Democratic Republic of Congo; 2 Centre de Recherche et d’Evaluation en Santé Globale et Action Humanitaire (CRESAH), Goma, Democratic Republic of Congo; 3 Faculty of Medicine and School of Public Health, Evangelical University in Africa (UEA), Bukavu, Democratic Republic of Congo; 4 Maternal and Infant Care Center, University of Washington Medical Center, Seattle, WA United States of America; 5 National Institute for Biomedical Research (INRB), Kinshasa, Democratic Republic of Congo; 6 Department of Medical Biology, School of Medicine, University of Kinshasa, Kinshasa, Democratic Republic of Congo; 7 Rodolphe Mérieux INRB-Goma Loaboratry, Goma, Democratic Republic of Congo; 8 Department of Internal Medicine, School of Medicine, University of Kisangani, Kisangani, Democratic Republic of Congo; 9 Ministry of Public Health, Hygiene and Prevention, Kinshasa, Democratic Republic of Congo; 10 Department of Surgery, School of Medicine, Official University of Bukavu, Bukavu, Democratic Republic of Congo; 11 Department of Obstetrics and Gynecology, School of Medicine, Official University of Bukavu, Bukavu, Democratic Republic of Congo; 12 Department of Obstetrics and Gynecology, School of Medicine, University of Kisangani, Kisangani, Democratic Republic of Congo; 13 Department of Specialities, School of Medicine, Official University of Bukavu, Bukavu, Democratic Republic of Congo; 14 Department of Pediatrics and Neonatology, School of Medicine, University of Goma, Goma, Democratic Republic of Congo; 15 Department of Obstetrics and Gynecology, School of Medicine, University of Kinshasa, Kinshasa, Democratic Republic of Congo; 16 Center for Humanitarian Health, Johns Hopkins Bloomberg School of Public Health, Baltimore, MD United States of America

**Keywords:** Ebola and pregnancy, Genital tract lesions, Post-partum hemorrhage and urinary fistula, Maternal death, Limited resources, DR Congo

## Abstract

**Background:**

Ebola Virus Disease (EVD) increases the risk for complications to a healthy pregnancy and delivery, notably post-partum hemorrhage. Additionally, successful management of post-partum hemorrhage and resource-intensive clinical interventions are difficult in the low-resourced setting of an Ebola Treatment Unit (ETU).

**Case presentation:**

This report describes the clinical course of a pregnant adolescent, gravida 2 para 1, with a history of delivery by cesarean section only 12 months before her second pregnancy. She was vaccinated with rVSV-ZEBOV following the death of a family member with confirmed EVD. She was admitted to the ETU 8 days after her vaccination, where a diagnosis of EVD in pregnancy was made, and her RT-PCR results on the blood sample showed a high viral load. At admission, she was 32 weeks pregnant. She was treated with mAb114, a neutralizing monoclonal antibody. In the first two days of her treatment, viral loads were measured, and they progressively declined after the third day of treatment. On day 3 of admission, following the failure of tocolysis, she delivered a preterm live infant. Her delivery was complicated by post-partum hemorrhage, two lateral cervical and vaginal mucosa lesions, with leakage of urine through the vagina, suggesting a genito-urinary fistula due to the extension of these lesions. A manual uterine exploration was required to remove placental debris with the administration of uterotonic drugs. Methylene blue instillation confirmed the vesicovaginal fistula. Due to a lack of obstetrical equipment in the ETU, the full extent of the cervical lesions could not be assessed nor repaired. Despite our resuscitation measures and mechanical cervical compression, she died 14 h after delivery of hemorrhagic shock complicated by coagulopathy with severe acute respiratory distress in the setting of acute EVD infection despite decreasing viral load.

**Conclusion:**

In cases of Ebola in pregnancy, not all maternal deaths due to post-partum hemorrhage are necessarily due to EVD and, therefore, could be averted with resources to provide specialized obstetric and post-partum care.

## Background

Ebola Virus Disease (EVD) is a zoonosis [1], caused by viruses of the genus *Orthoebolavirus* (family *Filoviridae*), including *Zaire ebolavirus* [2], the species first identified in the Democratic Republic of the Congo (DRC) in 1976 [3]. It is transmitted to humans by handling and/or eating certain infected wild animals such as bats, monkeys, gorillas, and apes. EVD is a highly contagious disease, and human-to-human transmission occurs through contact with body fluids from infected individuals or contaminated objects [1, 4, 5]. Clinically, EVD presents with severe symptoms, including fever, headache, fatigue, muscle pain, vomiting, diarrhea, and in severe cases, hemorrhagic manifestations, often resulting in high mortality [5]. From 2014 to 2016, the West African region experienced the deadliest outbreak recorded, with 11,310 deaths out of 28,616 cases registered [6]. The 10th outbreak followed this epidemic in the DRC (2018–2020), which is the second most deadly recorded, with 3470 probable and confirmed cases and a fatality rate of 66% [7]. For many contextual reasons, women are more exposed to infection than men, but vulnerability to the disease remains equal in both sexes [8]. Exacerbated by the natural physiological decrease in maternal immunity, pregnant women often experience obstetrical complications of EVD infection, including abortion, premature rupture of the membranes, premature delivery, fetal death in utero (FDIU), and hemorrhage during pregnancy and/or post-partum [9]. Maternal, fetal, and neonatal survival of infected women and their pregnancies are rare. A review of 12 studies revealed a maternal mortality rate of 84.3% out of 108 cases and a fetal mortality of 99.1% [10]. Maternal deaths in Ebola Treatment Units (ETUs) often result from post-partum hemorrhage, a post-delivery bleeding risk that, while not directly attributed to EVD infection, may be exacerbated by it [11]. Poor obstetric care can also expose women to obstetric fistulas [12]. Ebola outbreaks can compromise access to skilled obstetric care, as illustrated by our patient, thereby increasing the risk of obstetric fistula. According to the United Nations, an estimated 500,000 women currently live with obstetric fistula worldwide [13] and 50,000 -100,000 new cases occur each year, especially in countries with limited resources [14, 15]. Although many cases of spontaneous bladder rupture following vaginal delivery are documented [16], the same obstetrical complication cannot be ruled out during childbirth in an ETU. Understanding the diversity of etiologies that contribute to maternal deaths of women with EVD is constrained by various contextual factors. EVD outbreaks occur, in most cases, in remote areas where healthcare systems are weak, and patient treatment is provided under challenging conditions, especially when managing obstetrical and gynecological emergencies. During outbreaks in DRC, in some ETUs, medical teams lacked essential obstetrical equipment and medications [4, 17], challenging the management of obstetrical emergencies and leading to birth complications that could have been addressed with adequate supplies. This report describes a patient whose delivery was complicated by genital tract soft tissue lesions and urinary fistula who died in an under-resourced ETU during the 10th epidemic in the DRC. Her death could have been avoided with timely access to adequate obstetric and surgical care.

## Case presentation

During the 10th outbreak of Ebola in Eastern DRC, in our ETU, we admitted a pregnant adolescent, gravida 2 para 1 at 32 weeks pregnant and weighing 45 kg for suspected EVD. Upon admission, her clinical symptoms comprised fever, headache, nausea, chills, dizziness, joint and abdominal pain. Symptom onset occurred 7 days before her ETU consultation. The day following symptom onset, she consulted a local health center, where a diagnosis of malaria in pregnancy was confirmed with a rapid diagnostic test (RDT). She was treated with oral medications (artesunate-amodiaquine combination 100/270 mg twice daily for 3 days, amoxicillin 500 mg thrice daily for 10 days, and paracetamol 1 g thrice daily for 5 days) without symptomatic improvement, which motivated her transfer to the ETU to ensure proper care.

After being identified as a high-risk contact of a confirmed case who died from EVD infection, she was vaccinated against EVD by Ervebo^®^, rVSV-ZEBOV (Recombinant Vesicular Stomatitis Virus-Ebola Zaire, Merck, Philadelphia, USA) 4 days before symptom onset. During her admission evaluation, fetal movement was present. She was alert and oriented x4 but weak and lethargic, appeared in ill health, and was bedridden. She presented with fever (38.5 °C). Her other vital signs were within normal limits, with blood pressure (BP) of 126/62 mmHg, heart rate (HR) of 98 beats/minute, respiratory rate (RR) of 22 breaths/minute, and arterial oxygen saturation (SaO_2_) at 98% on free air.

The abdomen was enlarged by a gravid uterus with a median sub-umbilical scar; the fetal presentation was cephalic unfixed, and position II. Due to the lack of an ultrasound machine and Doppler fetoscope in the ETU, fetal heart tones were undetectable. Auscultation using a Pinard fetoscope is impossible while in full personal protection equipment required for work in the ETU. Every 10 min, we recorded 2-3 low-intensity uterine contractions of low intensity. The vulva examination was normal, and the cervix medial, dilated 2 cm, 50% effaced and the pelvis was borderline. Her blood group was O rhesus positive, hemoglobin 11 g/dL, hematocrit 38% and platelets 278 × 10^9^/L. We diagnosed an infectious syndrome (EVD and/or malaria) complicated by a risk of premature delivery. We confirmed malaria with plasmodium falciparum strain with a rapid diagnostic test (RDT). The patient received the supportive treatment by intravenous artesunate 2 × 108 mg/day for 4 days, paracetamol 2 × 1 g/day, tramadol 2 × 100 mg /day, papaverine 2 × 40 mg, multivitamins 3 × 1 tablet/day, omeprazole 2 × 40 mg/day, ceftriaxone 2 g/day, albendazole 1 × 400 mg one-time dose and oral rehydration solution (ORS). We were unable to administer steroids for fetal lung development in preparation for pre-term delivery due to a lack of dexamethasone in the ETU.

As she was admitted to the ETU in the evening, samples could only be run the following day due to the security risks of operating the laboratory in the evenings. Her real-time polymerase chain reaction (RT-PCR) analysis of blood samples confirmed Ebola infection through the GeneXpert^®^ (Cepheid, Sunnyvale, CA, USA), which revealed cycle threshold (Ct) values of glycoprotein (GP) 23.7 and nucleoprotein (NP) 19.3. According to the treatment protocol initiated by the PALM trial [18], all patients with confirmed EVD were entitled to receive one of the only two monoclonal antibody treatments available: a neutralizing monoclonal, REGN-EB3 (INMAZEB^®^, Regeneron Pharmaceuticals, US FDA, USA) and a neutralizing monoclonal antibody (mAb114), Ebanga^®^ (Ansuvimab, Ridgepack Biotherapeutics, USA). Per protocol, allocation was done by randomization [19]. Our patient was allocated to receive mAb114 and received a one-time intravenous infusion of 50 mg/kg/hour. Four hours after the monoclonal antibody infusion, her general condition deteriorated, and she began experiencing dyspnea and agitation. Her maximum temperature was measured at 40.1 °C, and her SaO_2_ was 88–98% under oxygen therapy. We noticed discrete breast tension and a decrease in active fetal movement. To manage her fever, we applied a wet wrap over her skin and administered antipyretics. We guided the patient through position changes to alleviate dyspnea and encourage fetal perfusion, alternating between the semi-seated position (for diaphragm decompression by the gravid uterus) and the left lateral decubitus position to maximize fetal perfusion. She remained under close observation for clinical improvement. Without ultrasound and fetal doppler, fetal heart rate could not be measured. The patient’s hemogram showed hemoglobin 10.8 g/dL, hematocrit 37%, and platelets 269 × 10^9^/L with no signs of hemolysis. Repeated RT-PCR results showed increased viral load with Ct values of GP 22.0 and NP 16.9 (Table 1).

**Table 1:** Viral load evolution, specific treatment, labor, fetal state and maternal outcome

Day	1		2		3		4	
Ct values (RT-PCR)	NP	19.3	NP	16.9	NP	19.7	NP	24.9
	GP	23.7	GP	22.0	GP	23.4	GP	29.3
Monoclonal anti-body (mAb114)	-		Perfusion of 50mg/kg/h (self-dose)		-		-	
Malaria test (RDT)	Positive		Positive		Positive		Negative	
Temperature (°C)	38.5		40.1		37.4		36.2	
Evolution of Labor	Uterine contractions with very low intensity		Non-effective uterine contractions		Effective contractions and spontaneous vaginal delivery; Uterine tonicity		Uterine tonicity with worsening PPH due to cervical and vaginal lesions	
Fetal state	Presence of normal Active fetal movements		Decrease of active fetal movements		Absence of fetal movement perceived		Premature live birth, and neonatal survival	
Breast state	Normal		Discrete breast tension		Sensitive breast tension, and bilateral galactorrhea		Decrease of breast tension, breast-milk flow	
Vaginal bleeding	None		None		Worsening PPH due to retention of placental debris, uterine atony and cervical vaginal lesions		Worsening PPH due to cervical and vaginal lesions and coagulopathy	
Involuntary loss of urine	None		None		Vaginal urine loss after delivery due to GUF		Vaginal urine loss due to GUF	
Maternal state evolution	Alive		Alive		Alive		Maternal death 14 hours following delivery	

Around 3 a.m. on the third day, following worsening clinical presentation and absence of active fetal movements, bilateral galactorrhea, and breast tenderness with increased tension, we suspected FDIU (Table 1). We could not confirm without ultrasound machine nor electronic fetoscope. Despite the concerning results of her most recent RT-PCR, asthenia, cough and low-intensity uterine contractions, our patient’s general clinical presentation slightly improved over the course of the morning. Her temperature returned to a normal range, between 36.2 and 37.4 °C. At 2 pm, a repeat RT-PCR showed a decreased Ebola viral load (Ct values of GP 23.40 and NP 19.7), while the malaria test remained positive (Table 1). Her uterine contractions were becoming effective with cervical dilation of 4 cm, effacement at 80%, with intact membranes and a fixed fetal presentation. Given her borderline pelvis and her history of a recent cesarean Sect.  (12 months before), delivery by cesarean section would have been the safest choice, but impossible in the setting of an ETU.

Around 9 p.m., she gave birth by vaginal delivery to a newborn premature female with Appearance-Pulse-Grimace-Activity-Respiration (APGAR) scores of 5 at the 5th minute, weighing 1850 g without apparent malformations. The umbilical cord was cut using sterile compresses and a sterile razor blade. Samples from the newborn’s buccal swab and venous whole blood tested negative for Ebola, confirming she was born uninfected. She subsequently received intravenous prophylactic INMAZEB^®^ (REGN-EB3) of 150 mg/kg and survived.

The delivery took place in the general hospitalization room for EVD-positive female patients on a standard hospital bed (180 × 90 cm) in the absence of a delivery room or delivery bed in the ETU. This restricted setting made it difficult for the patient to change position in labor and for the midwives to find space to intervene quickly. Delivery was immediately followed by moderate genital bleeding. In the third stage of labor, we treated the patient following Active Management of the Third Stage of Labor (AMTSL) guidance [20, 21]. She delivered her placenta within 4 min, but it was followed by significant post-partum hemorrhage. A urinary catheter was placed to empty the patient’s bladder; the urine was blood-tinged. We laboriously performed a manual uterine exploration with the extraction of minimal placental debris.

In addition to uterine massage, we started a slow infusion of 20 IU of oxytocin and gave 400 micrograms of misoprostol rectally. Within 10 min, the uterus had become tonic, palpated at the umbilicus with a decrease in bleeding to half. A transfusion of 450 mL of matched whole blood and perfusion of Ringers Lactate and glucose containing fluids were started.

During uterine exploration, we identified two lateral cervical lesions and lacerations of the posterior vaginal mucosa, the anterior vaginal mucosa could not be inspected. Her continued bleeding raised suspicion for cervical-vaginal artery lesions, and leakage of urine through the vagina, along with blood-tinged urine observed in the bladder catheter, indicated the possibility of a genito-urinary fistula. A methylene blue test performed via intravesical instillation was positive, confirming a vesico-vaginal fistula. However, the full extent of her soft tissue was impossible to explore or repair due to a lack of gynecological diagnostic equipment. Compressive gauze was placed successively into the vaginal canal to slow her bleeding without success. The compresses gauze were wholly soaked with bright red blood every 1–2 h.

At about 10 a.m. the next day, despite a negative malaria test, a progressive decrease in viral load measured (Ct values GP 29,3 and NP 24,9) that demonstrated good immune response and adequate uterine tonicity, the patient developed a coagulopathy complicating her hemorrhagic shock (Table 1). We noted agitation and altered mental status with vital sign changes BP (90/40–70/30 mm Hg), HR (126–148 beats/minute), RR (46 − 32 breaths/minute), Temperature (36.2–33.4 °C) and SaO_2_ (88 − 41%) under oxygen.

At 11.00 a.m., post-partum day one, she had been transfused with 3250 ml of blood from Ebola survivors and 6 L of fluids, calcium gluconate, and furosemide. The last control of hemoglobin and platelets were respectively low 3 g and 65% x 10^9^/L. At 11.45 a.m., i.e., around 14 h after delivery, she died of hemorrhagic shock complicated by coagulopathy with severe acute respiratory distress.

## Discussion

Our patient died of post-partum hemorrhage secondary to soft tissue cervical lesions, which occurred after a vaginal delivery on a borderline pelvis in the acute phase of EVD. However, her viral load had been gradually decreasing for two consecutive days, on her delivery date and the morning of her death. Malaria was detected on admission, indicating a transient co-infection. In pregnancy, malaria is associated with severe complications [22, 23], including maternal death, FDIU , anaemia, placental insufficiency, fetal growth restriction, and an increased risk of preterm birth, which may have contributed to her preterm delivery. Nevertheless, it did not appear to play a direct role in her death, as her malaria test on the day of death was negative. We found cervical and vaginal lesions during our clinical evaluation. Still, apart from bladder fistula, we could not specify the extent of the soft tissue cervical and vaginal lesions nor confirm nor treat this diagnosis due to a lack of the equipment needed to repair genital tract lesions and resuscitate hemorrhaging patients. Due to the limitations of our care setting and clinical supplies, we were unable to intervene further and watched helplessly as our patient’s state deteriorated until cardiac arrest.

This is not an isolated case of maternal death by post-partum hemorrhage (PPH) in the ETU; however, it remains the first case where the evaluation went so far as to identify the origin of the PPH with evidence of the cervical and vaginal lesions, as well as a vesicovaginal fistula observed. Many maternal deaths in ETUs are related to genital hemorrhage (post-partum or post-abortion), which, in the absence of diagnostics, can only be attributed to EVD-related coagulopathy [9]; yet it remains a very late complication of clinical decompensation [24]. We believe that the availability of gynecological diagnostics and repair in ETUs to direct early intervention could avert many of these deaths. This case also demonstrates that these deaths cannot necessarily be attributed to EVD alone.

According to the WHO, in 2020, about 800 women died every day from pregnancy-related causes [25]. PPH is the leading cause of these deaths. In ETUs, women giving birth are not immune to the risk of PPH and are likely at an increased risk [11]. ETUs should be equipped as other health facilities where women give birth, with all necessary equipment and supplies to ensure adequate management of related complications and strict compliance with Infection Prevention and Control (IPC) measures. The risk of bleeding and many other pregnancy complications is high during Ebola infection [9, 17]. While diagnosing PPH is not complex, it remains resource-intensive to determine its cause and provide effective treatment in ETUs. Although urinary fistula generally occurs in the middle post-partum period, spontaneous bladder rupture remains one of the rare documented complications following a vaginal delivery [16]. Repairing soft tissue injuries in the genital and urinary tract is very challenging with the limited resources in an ETU. Among the many causes of PPH, soft tissue injury represents 20% of cases, and uterine atony represents about 70% [26]. The rich and complex vascularity of the cervix and vaginal mucosa [27] require immediate repair of lesions. However, many ETUs lack the necessary obstetric/gynecologically trained providers, equipment, or delivery rooms [4, 17, 23], to provide essential emergency interventions. Furthermore, transferring a confirmed Ebola patient to a better-equipped community hospital was not feasible due to the high risk of disease transmission and infection control constraints. This case presented evidence of genito-urinary fistula, urine-saturated vaginal compresses gauze, and blood noted in the patient’s urine collection bag a methylene blue test was positive, confirming a vesicovaginal fistula. Still, its localization was impossible, given the limited resources in the ETU.

Generally, the risks of obstetric fistula include vaginal delivery on a borderline pelvis and mechanical dystocia and/or long labor [14, 28]. Our patient presented with both risk factors. In this case, the fistula must have been due to the direct extension of lesions of the soft tissues of the genital tract to her bladder. This type of injury could have been directly repaired if the conditions for emergency obstetrical care were met in the ETU. Given the dual vulnerability of women to both pregnancy-related complications and severe infections, we recommend maternal care conditions for pregnant women be improved in ETUs. Limited capacity to intervene promptly with the necessary clinical materials contributes to poor outcomes for EVD-positive PPH patients despite health workers’ best efforts [4, 17, 23]. We believe, as delivery by vacuum aspiration and uterine revision are now practiced in ETUs for EVD-confirmed women, cesarean section or repair of soft tissue injuries of the genital and urinary tract can also be practiced by experienced teams if all conditions are met for maintaining strict compliance with IPC measures. We believe this can be done with proper planning and investment. The cause of death for this patient was recorded as hemorrhagic shock due to cervical and vaginal lesions complicated by systemic coagulopathy. Still, we argue that EVD-induced coagulopathy may not have been the cause.

Before this patient’s delivery, her viral load was downtrending, and she was showing progressive clinical recovery from her EVD infection. We believe obstetric complications contributed to her death and preparation for their appropriate management should be considered in future clinical resource allocation and staffing decisions in ETUs. Obstetric dystocia and other complications remain a threat for pregnant women with EVD who must deliver in ETUs. We therefore strongly recommend ETUs be equipped with appropriate staff, infrastructure (operating room, delivery room) and equipment (delivery beds, operating tables, delivery kits, cesarean section kits, vaginal surgery kit, ultrasound equipment, doppler fetoscope, obstetrical monitoring equipment, with anesthetic products, anesthesia equipment, suture threads) to manage obstetrical complications and facilitate their implementation with adherence to the highest standards of IPC.

## Conclusion

In ETUs, not all maternal deaths from post-partum hemorrhage are due to Ebola infection. Inadequate obstetrical equipment and a lack of delivery and operating rooms equipped to manage soft tissue lesions after childbirth are contributing factors. The patient, with a borderline pelvis, a short interpregnancy interval, and failed tocolysis, was eligible for cesarean delivery. She underwent vaginal delivery complicated by soft-tissue lesions that resulted in a fistula and PPH, which could not be managed due to limited resources, ultimately leading to her death, despite a decreasing viral load. This case highlights the high risk of severe and potentially preventable obstetric complications in ETUs and underscores the need for improved clinical resources. We believe that with adequate emergency obstetric capabilities, this patient’s death could have been averted.

## Data Availability

The datasets used and/or analysed for the current study are available from the corresponding author upon request.

## Abbreviations

EVD: Ebola Virus Disease
ETU: Ebola Treatment Unit
PCR: Polymerase Chain Reaction
NP: Nucleoprotein
GP: Glycoprotein
rVSV-ZEBOV: Recombinant Vesicular Stomatitis Virus-Ebola Zaire
AMTSL: Active Management of the Third Stage of Labour
APGAR: Appearance, Pulse, Grimace, Activity, Respiration
IPC: Infection Prevention and Control
FDIU: Fetal death in utero
PPH: Post-partum Hemorrhage
WA: Week of amenorrhea
WS: Week of gestation
BP: Blood pressure
HR: Heart rate
HR: Respiratory rate
T°: Temperature
SaO_2_: Arterial oxygen saturation
Mab114: Neutralizing monoclonal antibody
GUF: Genital urinary fistula
US FDA: United States Food and Drug Administration
MOH: Ministry of Health

## References

[1] Gumusova S, Sunbul M, Leblebicioglu H. Ebola virus disease and the veterinary perspective. BioMed Cent Ltd. 2015. 10.1186/s12941-015-0089-x. 10.1186/s12941-015-0089-x PMC4450609 26018030

[2] International Committee on Taxonomy of Viruses. Virus Taxonomy: Filoviridae: Orthoebolavirus. ICTV Report. Accessed 30 Nov 2025. https://ictv.global/report/chapter/filoviridae/filoviridae/orthoebolavirus.

[3] Centers for Disease Control and Prevention. Outbreak History. Ebola Disease Outbreaks by Species and Size, Since 1976. https://www.cdc.gov/ebola/outbreaks/index.html. Accessed November 26, 2025.

[4] Imani-musimwa P, Grant E, Mukadi-Bamuleka D, Fraterne-Muhayangabo R, Kitenge-Omasumbu R, Mbala-Kingebeni P, et al. Neonatal Survival Following Spontaneous Maternal Recovery From Ebola Virus Disease in a Resource-Limited Setting in Western Democratic Republic of the Congo, 2025; 2025. 10.1155/crdi/2987569. 10.1155/crdi/2987569 PMC11928211 40125488

[5] World Health Organization (WHO). Ebola virus disease. https://www.who.int/news-room/fact-sheets/detail/ebola-disease. Accessed November 28, 2025.

[6] Centers for Disease Control and Prevention. 2014–2016 Ebola Outbreak Distribution in West Africa. Accessed April 20, 2023. https://www.cdc.gov/vhf/ebola/history/2014-2016-outbreak/distribution-map.html

[7] World Health Organization. Democratic Republic of Congo. Rapport technique: Revue après action de la riposte aux 9e, 10e, 11e et 12e épidémies de maladie à virus Ebola. Kinshasa, Juin, 2021.

[8] Nkangu MN, Olatunde OA, Yaya S. The perspective of gender on the Ebola virus using a risk management and population health framework: A scoping review. BioMed Cent Ltd. 2017. 10.1186/s40249-017-0346-7. 10.1186/s40249-017-0346-7 PMC5635524 29017587

[9] World Health Organization (WHO). Guidelines for the management of pregnant and breastfeeding women in the context of Ebola virus disease., https://www.who.int/publications/i/item/978924000138. Accessed September 22, 2024. 32091684

[10] Garba I, Dattijo L, Habib A. Ebola virus disease and pregnancy outcome: A review of the literature. Trop J Obstet Gynaecol. 2017;34(1). 10.4103/tjog.tjog_3_17.

[11] Centers for Disease Control and Prevention. Guidance for Screening and Caring for People who are Pregnant with Ebola Virus Disease for Healthcare Providers in U.S. Hospitals. https://www.cdc.gov/vhf/ebola/clinicians/evd/pregnant-women.html. Accessed 6 May 2024.

[12] Mukwege D. ‘Fistula Should Not Happen,’ Fistula Foundation. Accessed November 20, 2025. https://fistulafoundation.org/news/fistula-should-not-happen/?utm_source=chatgpt.com

[13] Nations U, United Nations Population Fund (UNFPA). ‘A silent crisis’: Obstetric fistula affects 500,000 women, yet it’s fully treatable. https://www.who.int/news-room/facts-in-pictures/detail/10-facts-on-obstetric-fistula. Accessed 15 Nov 2025.

[14] United Nations Population Fund (UNFPA). Quand l’accouchement nuit à la santé: La fistule obstétricale. https://www.unfpa.org/sites/default/files/resource-pdf/FR-SRH%20fact%20sheet-Fistula.pdf. Accessed April 12, 2024.

[15] World Health Organization (WHO). Obstetric fistula. https://www.who.int/news-room/facts-in-pictures/detail/10-facts-on-obstetric-fistula. Accessed November 20, 2025.

[16] Stabile G et al. Spontaneous bladder rupture after normal vaginal delivery: Description of a rare complication and systematic review of the literature, *Multidisciplinary Digital Publishing Institute (MDPI)*. 10.3390/diagnostics11101885. Oct. 01, 2021. 10.3390/diagnostics11101885 PMC8534473 34679583

[17] Imani-Musimwa P, Grant E, Feza-Malira M, Mbala-Kingebeni P, Buhoro-Baabo G, Zawadi-Endanda E, et al. A premature newborn born to an adolescent girl with acute Ebola virus disease and malaria survives in a resource‐limited setting in an Ebola treatment unit in DR Congo: ‘A case report’. Clin Case Rep. 2023;11(11). 10.1002/ccr3.8253. 10.1002/ccr3.8253 PMC10665585 38028078

[18] Mulangu S, Dodd L, Davey Jr RT, Mbaya OT, Proschan M, Mukadi D, et al. A Randomized, Controlled Trial of Ebola Virus Disease Therapeutics. N Engl J Med. 2019;381(24):2293–303. 10.1056/nejmoa1910993. 10.1056/NEJMoa1910993 PMC10680050 31774950

[19] National Institutes of Health (NIH). Independent monitoring board recommends early termination of Ebola therapeutics trial in DRC because of favorable results with two of four candidates. https://www.nih.gov/news-events/news-releases/independent-monitoring-board-recommends-early-termination-ebola-therapeutics-trial-drc-because-favorable-results-two-four-candidates. Accessed September 11, 2022.

[20] The Global Library of Women’s Medicine (GLOWM). The Active Management of the Third Stage of Labor. https://www.glowm.com/pdf/amtsl_wallchart_single_final.pdf. Accessed July 15, 2023.

[21] Begley CM, Gyte GML, Devane D, McGuire W, Weeks A, Biesty LM. Active versus expectant management for women in the third stage of labour, *John Wiley and Sons Ltd*. 10.1002/14651858.CD007412.pub5. Feb. 13, 2019. 10.1002/14651858.CD007412.pub5 PMC6372362 30754073

[22] Waxman M, Aluisio AR, Rege S, Levine AC. Characteristics and survival of patients with Ebola virus infection, malaria, or both in Sierra Leone: a retrospective cohort study. Lancet Infect Dis. Jun. 2017;17(6):654–60. 10.1016/S1473-3099(17)30112-3. 10.1016/S1473-3099(17)30112-3 28258817

[23] Imani-Musimwa P, et al. Maternal and fetal survival following Ebola, HIV and Malaria co-infection in the first trimester of gestation in resource-limited setting in Democratic Republic of Congo. BMC Pregnancy Childbirth. 2025;25(1):210. 10.1186/s12884-025-07265-0. 10.1186/s12884-025-07265-0 PMC11863912 40011840

[24] Takeda J, Takeda S. Management of disseminated intravascular coagulation associated with placental abruption and measures to improve outcomes. Korean Soc Obstet Gynecol. 2019. 10.5468/ogs.2019.62.5.299. 10.5468/ogs.2019.62.5.299 PMC6737058 31538072

[25] WHO, UNICEF, UNFPA, World Bank Group, UNDESA. Trends in maternal mortality 2000 to 2020. https://iris.who.int/bitstream/handle/10665/366225/9789240068759-eng.pdf?sequence=1. Accessed 12 Jan 2024.

[26] Bienstock JL, Eke AC, Hueppchen NA. Postpartum Hemorrhage, *N. Engl. J. Med.*, 2021; 384(17): 1635–1645. 10.1056/NEJMra1513247. 10.1056/NEJMra1513247 PMC10181876 33913640

[27] Bereza T, Tomaszewski KA, Bałajewicz-Nowak M, Mizia E, Pasternak A, Walocha J. The vascular architecture of the supravaginal and vaginal parts of the human uterine cervix: A study using corrosion casting and scanning electron microscopy, *J. Anat.*, 2012;221(4): 352–357. 10.1111/j.1469-7580.2012.01550.x. 10.1111/j.1469-7580.2012.01550.x PMC3458254 22844876

[28] United Nations Population Fund (UNFPA). Obstetric fistula. https://www.unfpa.org/obstetric-fistula#readmore-expand. Accessed April 12, 2024.

